# Elevated Small Nuclear Ribonucleoprotein Polypeptide an Expression Correlated With Poor Prognosis and Immune Infiltrates in Patients With Hepatocellular Carcinoma

**DOI:** 10.3389/fonc.2022.893107

**Published:** 2022-07-04

**Authors:** Youfu Zhang, Xuyang Wang, Huaxiang Wang, Yi Jiang, Zhidan Xu, Laibang Luo

**Affiliations:** ^1^ Department of Organ Transplantation, Jiangxi Provincial People’s Hospital, The First Affiliated Hospital of Nanchang Medical College, Nanchang, China; ^2^ Department of Hepatobiliary Surgery, The Fuzong Clinical Medical College of Fujian Medical University, Fuzhou, China; ^3^ Department of Hepatobiliary Surgery, 900 Hospital of the Joint Logistic Team, Fuzhou, China

**Keywords:** SNRPA, prognostic value, hepatocellular carcinoma, immune infiltrates, spliceosome

## Abstract

**Background:**

Elevated Small Nuclear Ribonucleoprotein Polypeptide A (*SNRPA*) can enhance tumor cell growth and proliferation in various cancers. However, rarely studies focus on the comprehensive analysis of *SNRPA* in hepatocellular carcinoma (HCC).

**Methods:**

TCGA and GEO databases were used to analyze the mRNA expression of *SNRPA* in HCC. Protein expression of SNAPA was validated using immunohistochemistry. Stably transfected HCC cells were used to investigate the role of *SNRPA* in the progression of HCC. The functional enrichment analysis was utilized for the biological function prediction. The CIBERSORT and ssGSEA algorithms were used to evaluate the composition of the tumor microenvironment and immunocyte infiltration ratio.

**Results:**

The *SNRPA* expression was upregulated in HCC and positively correlated with tumor stage and grade. *SNRPA* overexpression were independent risk factors for poor overall survival (OS) and recurrence-free survival (RFS). In patients with early-stage disease, low alpha-fetoprotein expression, and better differentiation, *SNRPA* still exhibited the excellent prognostic value. Knockdown of *SNRPA* inhibited the proliferation and migration while promoting the apoptosis of HCC cells. Higher methylation of the CpG site cg16596691 correlated with longer OS in HCC patients. Genes co-expressed with *SNRPA* were overexpressed in HCC and correlated with shorter OS. The GO and KEGG enrichment analysis showed that *SNRPA* expression was related to mRNA splicing, spliceosome signaling. GSEA demonstrated that the main enrichment pathway of SNRPA-related differential genes was spliceosome signaling, cell cycle signaling, P53 signaling pathway, T cell receptor signaling pathway, natural killer cell-mediated signaling. CIBERSORT and ssGSEA algorithm revealed that *SNRPA* was mainly associated with the higher proportion of CD8+T cells, T cells follicular helper, T cells regulatory, Macrophages M0, and the lower proportion of T cells CD4 memory resting, NK cells resting, Monocytes, and Mast cells resting.

**Conclusion:**

Elevated *SNRPA* enhances tumor cell proliferation and correlated with poor prognosis and immune infiltrates in patients with HCC.

## Introduction

Hepatocellular carcinoma (HCC), the most common primary liver malignant, is the fifth leading cause of cancer-related death worldwide (1). Although significant progress has been made in cancer-related treatment technologies in recent years, the five-year survival rate is still less than 20% in all stages (2). The low diagnostic rate in the early stage of disease and high recurrence rate after curative hepatectomy have been mainly responsible for the poor prognosis (3, 4). Currently, early screening of HCC mainly relied on liver ultrasound examination and serum alpha-fetoprotein (AFP) analysis, but both techniques lack sufficient sensitivity for detecting the early lesions (5, 6). Therefore, the identification of diagnosis and prognosis-related novel molecular markers is crucial for providing novel clues of early diagnosis, guiding early treatment, and ameliorating the prognosis of patients with HCC (7).

The splicing process of pre-messenger RNA was essential in gene expression in eukaryotic cells (8, 9). The dysregulation of the alternative splicing process, such as aberrant expression of genes encoding the spliceosomal members and the delocalization of snRNPs, have now been proved to be the molecular basis of many diseases (10–12). small nuclear ribonucleoprotein polypeptide A (SNRPA) was one of the core proteins of the spliceosome. High SNRPA levels have been detected in varieties of cancers and associated with patients’ poor prognoses, such as gastric cancer, and colorectal cancer (13–15). Using bioinformatic analyses, SNRPA has been found overexpressed in HCC, but the prognostic significance and biological function has not yet been elucidated (16).

In this present study, we investigated the *SNRPA* mRNA and protein expression in HCC and adjacent normal tissues to uncover the diagnostic value, especially for the early stage of diseases. We then analyzed the association between *SNRPA* expression with the clinical characteristics and clinical outcomes. We conducted cellular and molecular biology assays to further investigate the role of SNRPA in the progression of HCC. Next, we queried the CpG sites and analyzed the correlation between SNRPA methylation with overall survival. The Gene Ontology (GO), Kyoto Encyclopedia of Genes and Genomes (KEGG) and Gene Set Enrichment Analyses (GSEA) were employed to identify the biological functions and signaling pathways through which SNRPA could involve in the tumorigenesis and progression of HCC. Finally, we investigated the correlation of SNRPA expression with the composition of the tumor microenvironment and immunocyte infiltration ratio by the CIBERSORT algorithm and ssGSEA algorithm.

## Materials and Methods

### *SNRPA* Expression Analysis in Various Public Databases

The mRNA expression level of *SNRPA* in HCC and adjacent normal tissues were analyzed using datasets from The Cancer Genome Atlas (TCGA) and the Gene Expression Omnibus (GEO) (GSE54236 and GSE76427 datasets) database. The Kaplan Meier plotter database was used to investigate the association between *SNRPA* expression with overall survival and recurrence-free survival.

### Patients and HCC Tissue Specimens

We performed immunohistochemical staining assay to investigate the prognostic significance of SNRPA protein expression in HCC. 161 HCC specimens and relevant complete clinicopathologic characteristics from the patients undergoing radical tumor resection from January 2012 to May 2014 at 900 Hospital of the Joint Logistics Team were collected. All HCC specimens were stored by formalin-fixed paraffin-embedded blocks. The complete clinicopathologic characteristics, including basic clinical features and the survival information. Survival data were acquired through repeat admissions, telephone follow-up, and the Social Security Death Index. The latest end of follow-up date for the last patient was Dec 21, 2019. Our inclusion criteria were listed as follows: patients underwent open liver resection, only one tumor lesion or multiple lesions but limited to one hepatic lobe, Child-Pugh class A or B, without any history of cancer treatment prior to hepatectomy, pathologic examination confirmed the diagnosis of HCC. This investigation was conducted in accordance with the principles of the Declaration of Helsinki, and approved by the Human Research Ethics Committee of the 900th Hospital of the Joint Logistics Team (Fuzhou, China). Written informed consent was obtained from all patients before surgery.

### Immunohistochemistry Assay

The formalin-fixed paraffin-embedded blocks were cut into 4-μm sections. Then, the sections were degreased and rehydrated using different concentrations of malondialdehyde and ethanol. The antigen retrieval used Tris/Ethylenediaminetetraacetic acid (EDTA) (pH 9.0) by boiling tissue sections for 20 min. The sections were co-incubated with 3% H2O2 for 10 min to eliminate endogenous peroxidase activity. Then, the sections were incubated with Anti-U1A antibody (ab155054; 1:300; Abcam, UK) for one hour at 25 °C. Next, the sections were incubated with secondary antibody (1:50,000; KIT-5010; anti-rabbit/mouse IgG; China Fuzhou Maixin Biotechnology Development Co., Ltd.) for 40 min at 25°C. Finally, the sections were stained with 3,3’-diaminobenzidine and substrate chromogen (Dako) and then counterstained with hematoxylin. SNRPA protein expression was assessed on a semi-quantitative IHC scoring system with five-point scale: 0 (no positive cells); 1 (less than 25% positive cells); 2 (26-50% positive cells); 3 (51-75% positive cells) and 4 (more than 75% positive cells). The IHC staining was evaluated by two separate experienced pathologists who were blinded to patients’ clinical information.

### Cell Culture and Plasmid Transfection

The hepatocellular carcinoma cell lines Huh7, HepG2, Hep3B, and normal hepatocyte cell line LO2 were purchased from the Shanghai Cell Bank of the Chinese Academy of Sciences (Shanghai, China). These cell lines were cultured in DMEM basal medium (Hyclone, SH30022.01) supplemented with 1% penicillin-streptomycin (Hyclone, SV30010) and 10% fetal bovine serum (FBS, Gibco, 10099141), in an incubator under a moist atmosphere of 5% CO2 at 37°C. The Huh7 and HepG2 cells were transfected with shSNRPA or sh-NC using Lipofectamine™ 3000 Transfection Kit (L3000015, Invitrogen, USA), according to the manufacturer’s protocol. After incubation for 48h at 37°C, cells were used for subsequent experiments.

### Quantitative Polymerase Chain Reaction (qRT-PCR)

The total RNA was extracted from the cells using RNAiso Plus (TaKaRa, 9109, China) and then reverse transcribed into cDNA for subsequent PCR assay using gDNA Purge (Novoprotein, E047-01A, China), according to the manufacturer’s instruction. Then, NovoStart^®^ SYBR qPCR SuperMix Plus (Novoprotein, E096-01B, China) and 7300 Real-Time PCR System (Applied Biosystems, USA) were used to perform the RT-qPCR. GAPDH was used as the internal control for *SNRPA*. The 2-ΔΔCt method was used to calculate the mRNA expression of SNRPA. The primer pairs of SNRPA and GAPDH were designed as following: SNRPA Forward: 5’-CAAACCTATGCGTATCCAGT-3’, Reverse: 5′‐GGATTCTCAGAAAGAGGCTG-3’ and GAPDH Forward:5’-ATGGGGAAGGTGAAGGTCG-3’, Reverse: 5’-TCGGGGTCATTGATGGCAACAATA-3’.

### CCK-8 Assay

After transfected 48 h, the HepG2 and Huh7 cells were harvested and seeded (8.0×104 cells/ml) into a 96-well microplate and then placed into an incubator at 37°C for 24, 48,72 and 96 h. Next, a 10 µl Cell Counting Kit-8 (CCK-8) (MA0218, Meilune, Dalian, China) reagent was added to each well for 90 min. The viable cells were determined using absorbance at a 450-nm wavelength.

### Transwell Migration and Invasion Assays

The transwell assays were performed using 24-well transwell plates (Corning Inc., Corning, NY, USA) to assess the migration and invasion abilities of HepG2 and Huh7 cells. For migration assays, 2 × 105 HCC cells per well were seeded in 200 µl of the serum-free medium in the upper chamber, and 700 µl of 15% FBS medium was added to the lower chamber serving as the chemoattractant solution. For transwell invasion assays, we precoated the 24-well transwell plates with 60µl 1:8 DMEM-diluted Matrigel (BD Biosciences, USA) for 2 h at 37°C before cells were seeded. Subsequently, the migrating and invading cells under the surface of the membrane were fixed by 4% paraformaldehyde (Aladdin, Shanghai, China) for 15 min and stained by crystal violet (MA0150, Meilune, Dalian, China).

### Cell Cycle

After cultured stable HepG2 and Huh7 cells, logarithmic-growth phase cells were digested by trypsinization and isolated. Cells were fixed with 75% ethanol at 4°C overnight. Then, 5% propidium iodide (PI) was used to stain the pelleted cells. Flow cytometry was used to determine the cell cycle distribution (MA0334, Meilune, Dalian, China). The Flojo software (Becton Dickinson, USA) was employed to calculate the percentages of HCC cells at different cell cycle stages.

### Cell Apoptosis

After being stably cultured, cells were digested by trypsinization (without EDTA), resuspended in 1× binding buffer at 1×106 cells/mL. Then, 5μl Annexin VFITC and 10μl PI (MA0220, Meilune, Dalian, China) were sequentially added to the cells. We determine the cell apoptosis using flow cytometry after the cells were incubated at 25°C for 15 min in the dark. Cells that Annexin V-FITC (–)/PI (–) were considered living cells. Cells that Annexin V-FITC (+)/PI (–) and Annexin V-FITC (+)/PI (+) were considered as early apoptotic cells and late apoptotic cells, respectively.

### DNA Methylation of *SNRPA* in HCC

We downloaded the RNAseq and Illumina Human Methylation 450 datasets from the UCSC Xena database to analyze the correlation between *SNAPA* expression and DNA methylation. Then, we identified the CpG sites that correlated with mRNA expression and survival rate from the MethSurv web tool (17).

### Co-Expressed Genes Analysis of *SNRPA*

Correlated genes that influence *SNRPA* mRNA expression were identified from the cBioPortal, LinkedOmics, and GEPIA databases, respectively. Then, overlapping genes from three databases with Pearson’s correlation values greater than 0.6 were considered as the co-expressed genes of *SNRPA*. Next, we performed the GO and KEGG enrichment analysis on these co-expressed genes in the DAVID database to explore the potential biological process and pathway that *SNRPA* regulates tumorigenesis and progression. In addition, a PPI network from the co-expressed genes was constructed in the STRING database and visualized in the Cytoscape software (Version 3.5.1).

### GSEA

We downloaded the RNAseq dataset of 373 HCC samples from TCGA and separated into high and low expression groups based on the median mRNA expression value of *SNRPA*. We then performed the GSEA enrichment analysis using GSEA software (Version 4.1.2). In this process, “c2.cp.kegg.v7.0.symbols.gmt” was selected as the functional gene set, and P-values <0.05 and false discovery rate q-values <0.25 were considered statistically significant.

### Immune Infiltration Analysis

The CIBERSORT software was used to calculate the proportion of 22 types of immune cells in each sample, and the single sample GSEA (ssGSEA) method with the “GSVA” R package was used to determine the level of tumor immune infiltration. We then investigated the correlations between *SNRPA* mRNA expression and enrichment of 22 immune cell types.

### Statistical Analysis

The R software with packages was utilized to perform statistical analysis and generate the figures. The Fisher exact test, two-tailed Student’s t-tests or Wilcoxon test were employed to analyze the associations between SNRPA expression and clinicopathologic features. The univariate and multivariate Cox regression analysis was employed to determine the prognostic value of SNRPA. Correlations analyses were performed using Spearman correlation tests. Kaplan-Meier method with log-rank test was used for comparison of survival rate. Receiver operating characteristic (ROC) curves and area under the curve (AUC) were performed to evaluate the diagnostic value of SNRPA in HCC. P < 0.05 was considered statistically significant.

## Results

### *SNRPA* mRNA Was Overexpressed and Associated With Poor Prognosis in Patients With HCC

**Figure 1:**
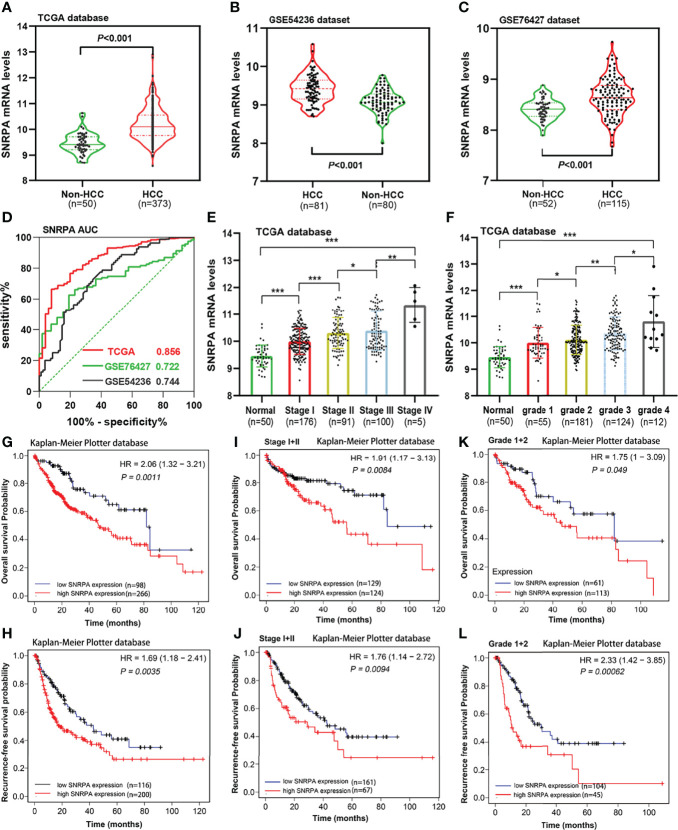
*SNRPA* mRNA levels in HCC and adjacent normal liver tissues and its prognostic value. **(A–C)** *SNRPA* mRNA levels were significantly greater in HCC than in normal liver tissues in the TCGA **(A)**, GSE54236 **(B)** and GSE76427 **(C)** datasets. **(D)** ROC curve exhibited the excellent diagnostic value of *SNRPA* mRNA for HCC in TCGA and GEO databases. **(E, F)** The *SNRPA* mRNA expression gradually increased with the tumor stage **(E)** and tumor grade **(F)** increased. **(G, H)** Higher *SNRPA* mRNA expression was associated with worse OS **(G)** and RFS **(H)**. **(I, J)** In patients with stage I+II, higher *SNRPA* mRNA levels correlated with poor OS **(I)** and RFS **(J)**. **(K, L)** In patients with grade 1 + 2, higher *SNRPA* mRNA levels correlated with poor OS **(K)** and RFS **(L)**. *P < 0.05, **P < 0.01, ***P < 0.001

We investigated the *SNRPA* mRNA levels in HCC tissues from TCGA and GEO (GSE54236 and GSE76427 datasets) database and found that *SNRPA* mRNA expression was significantly upregulated in HCC compared with adjacent normal liver tissues (all P<0.001, **Figures 1A–C**). In addition, ROC curves revealed that *SNRPA* mRNA level has excellent diagnostic significance (**Figure 1D**), with AUC values of 0.856 for TCGA, 0.722 for GSE76427, 0.744 for GSE54236. Furthermore, the expression of *SNRPA* mRNA gradually increased with the tumor stage (**Figure 1E**) and tumor grade (**Figure 1F**) increased. We next looked at the prognostic significance of *SNRPA* mRNA level and found that patients with high *SNRPA* expression have shorter OS and RFS (**Figures 1G, H**). As shown in **Figures 1I–L**, in patients with early tumor stages (stage I+II) and grades (grade I+II), high *SNRPA* mRNA levels also correlated with poorer OS and RFS (**Figures 1I–L**).

### SNRPA Protein Was Overexpressed and Associated With Poor Prognosis in a Cohort of 161 HCC Patients

**Figure 2:**
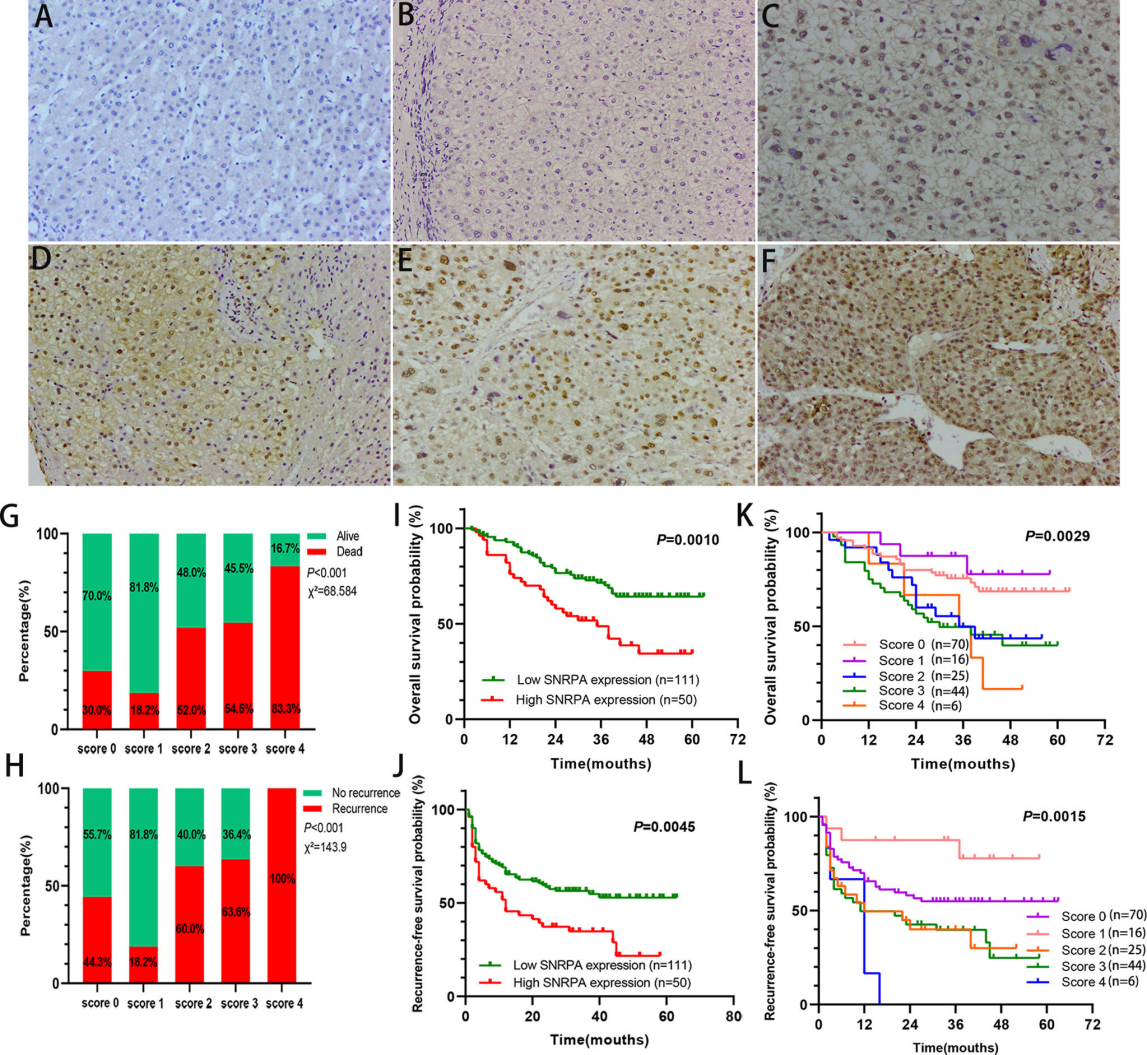
SNRPA protein levels in normal liver tissues and HCC tissues and its prognostic value. **(A)** Representative images of SNRPA IHC staining for normal liver tissues. **(B–F)** Representative images of SNRPA IHC staining for score of 0 **(B)**, 1 **(C)**, 2 **(D)**, 3 **(E)**, and 4 **(F)**, respectively in HCC tissues. **(G)** The percentage of alive and death in patients with different SNRPA IHC staining score. **(H)** The percentage of recurrence in patients with different SNRPA IHC staining score. **(I–J)** Higher SNRPA protein expression was associated with worse OS **(I)** and RFS **(J)**. **(K, L)** Kaplan–Meier curves of OS **(K)** and RFS **(L)** among patients with the different SNRPA IHC staining score.

The IHC staining demonstrated that SNRPA protein was predominantly seen in the nucleus. We exhibited the representative IHC staining pictures of SNRPA protein expression in adjacent normal liver tissue and different IHC scored HCC samples (**Figures 2A–F**). At the last patient follow-up, the death rates of patients with IHC staining score of 0, 1, 2, 3, and 4 were 30.0%, 18.2%, 52.0%, 54.5, and 83.3%, and the recurrence rates were 44.3%, 18.2%, 60.0%, 63.6% and 100%, respectively (**Figures 2G, H**). We divided the 161 HCC patients into two groups of high SNRPA expression (A score of 3 and 4, n=50) and low SNRPA expression (A score of 0, 1, and 2, n=111) based on the IHC staining score. We then investigated the correlations between SNRPA protein expression with clinicopathologic features in HCC. The results showed that patients with high SNRPA protein expression were found to be positively correlated with worse TNM stage (P=0.049), low tumor differentiation (P=0.009), vascular invasion (P=0.006), high recurrence rate (P=0.005), and high death rate (P=0.003), whereas not correlated with age, gender, tumor size, serum AFP level, tumor location, HBsAg, Edmonson grade, Child-Pugh class, and tumor encapsulation (**Table 1**). In addition, univariate Cox regression analysis indicated that high SNRPA protein level was one of the prognostic factors for OS and/or RFS in the HCC cohort (**Table 2**). The multivariate Cox regression analysis validated that high SNRPA protein level was an independent risk factor for OS ((aHR (95%CI) 1.724(1.011-2.939), P = 0.045) and/or RFS (aHR (95%CI) 1.672(1.062-2.631), P = 0.026) in the HCC cohort (**Table 3**). Kaplan-Meier curve revealed that high expression of SNRPA protein correlated with poorer OS and RFS in HCC (**Figures 2I-J**). Furthermore, there was an increasing trend towards poor OS and RFS with a higher IHC staining score (**Figures 2K-L**).

**Table 1:** Correlation between SNRPA protein expression and clinicopathologic features in 161 patients with hepatocellular carcinoma.

Characteristics			SNRPA level		χ²	*P-Value
		N	high(n)	low(n)		
Age (year)	>55	105	36	69	1.471	0.225
	<=55	56	14	42		
Gender	Male	141	44	97	0.012	0.973
	Female	20	6	14		
Tumor size (cm)	>5cm	86	32	54	3.265	0.071
	<=5cm	75	18	57		
TNM stage	I/II	107	28	79	3.559	**0.049**
	III	54	22	32		
Serum AFP level	>400ng/ml	66	20	46	0.030	0.863
	<=400ng/ml	95	30	65		
Tumor location	Left	53	16	37	0.028	0.868
	Right	108	34	74		
Tumor differentiation	Low	23	13	10	9.392	**0.009**
	Median	106	31	75		
	High	32	6	26		
HBsAg	Positive	74	21	53	0.459	0.498
	Negative	87	29	58		
Edmonson grade	I	28	10	18	0.344	0.558
	II-IV	133	40	93		
Child-Pugh class	A	76	27	49	1.344	0.246
	B	85	23	62		
Vascular invasion	Yes	77	32	45	7.603	**0.006**
	No	84	18	66		
Tumor encapsulation	Yes	110	35	75	0.094	0.759
	No	51	15	36		
Recurrence	Yes	83	34	49	7.855	**0.005**
	No	78	16	62		
Survival status	Alive	66	29	37	8.671	**0.003**
	Dead	95	21	74		

TNM, tumor node metastasis; AFP, alpha fetoprotein. *P-Value<0.05 were considered statistically significant. Bold value considered statistically significant.

**Table 2:** Univariate Cox Regression analysis of overall survival and recurrence-free survival in 161 patients with hepatocellular carcinoma.

variables		Overall survival	*P-Value	Recurrence-free survival	*P-Value
		HR (95%CI)	HR (95%CI)		
Age (year)	>55 vs. <=55	0.883(0.536-1.454)	0.625	0.704(0.453-1.095)	0.120
Gender	Male vs. female	0.664(0.285-1.549)	0.343	1.277(0.693-2.355)	0.433
Tumor size (cm)	>5 vs. <=5	1.934(1.156-3.234)	**0.012**	1.801(1.153-2.812)	**0.010**
TNM stage	I/II vs. III	1.935(1.188-3.152)	**0.008**	0.818(0.521-1.285)	0.383
Serum AFP level	>400 vs <=400	1.766(1.085-2.875)	**0.022**	1.597(1.037-2.458)	**0.033**
Tumor location	Left vs. right	0.851(0.513-1.414)	0.534	1.169(0.731-1.868)	0.515
Tumor differentiation	Hihg vs. median/low	1.347(0.686-2.644)	0.386	1.773(1.001-3.155)	**0.048**
HBsAg	Positive vs. negative	1.325(0.814-2.155)	0.257	0.952(0.617-1.469)	0.826
Edmonson grade	I vs. II-IV	0.640(0.305-1.341)	0.237	0.581(0.300-1.127)	0.108
Child-Pugh class	A vs. B	5.187(2.693-9.078)	**<0.001**	1.767(1.142-2.734)	**0.011**
Vascular invasion	Yes vs. no	1.859(1.131-3.054)	**0.014**	2.470(1.578-3.865)	**<0.001**
Tumor encapsulation	Yes vs. no	0.897(0.536-1.501)	0.679	0.288(0.185-0.447)	**<0.001**
SNRPA protein level	High vs. low	2.221(1.360-3.627)	**0.001**	1.840(1.187-2.853)	**0.006**

HR, Hazard ratio; CI, confidential interval; TNM, tumor node metastasis; AFP, alpha fetoprotein. *P-Value<0.05 were considered statistically significant. Bold value considered statistically significant.

**Table 3:** Multivariate Cox Regression analysis of overall survival and recurrence-free survival in 161 patients with hepatocellular carcinoma.

variables		Overall survival	*P-Value	Recurrence-free survival	*P-Value
		aHR (95%CI)	aHR (95%CI)		
Tumor size (cm)	>5 vs. <=5	0.971(0.501-1.884)	0.931	0.814(0.498-1.329)	0.411
TNM staging	I/II vs. III	0.879(0.472-1.634)	0.682		
Serum AFP level	>400 vs <=400	0.649(0.389-1.084)	0.099	0.682(0.434-1.072)	0.097
Tumor differentiation	Hihg vs. median/low			0.912(0.478-1.740)	0.780
Child-Pugh class	A vs. B	0.192(0.106-0.349)	**<0.001**	0.723(0.456-1.148)	0.169
Vascular invasion	Yes vs. no	0.642(0.372-1.106)	0.110	0.478(0.293-0.781)	**0.003**
Tumor encapsulation	Yes vs. no	0.808(0.476-1.372)	0.431	3.433(2.163-5.448)	**<0.001**
SNRPA protein level	High vs. low	1.724(1.011-2.939)	**0.045**	1.672(1.062-2.631)	**0.026**

aHR, adjusted Hazard ratio; CI, confidential interval; TNM, tumor node metastasis; AFP, alpha fetoprotein. *P-Value<0.05 were considered statistically significant. Bold value considered statistically significant.

### SNRPA Protein Expression Has Prognostic Significance in Patients With Early-Stage, Low Tumor Grade, and Median/High Differentiation Subgroups

**Figure 3:**
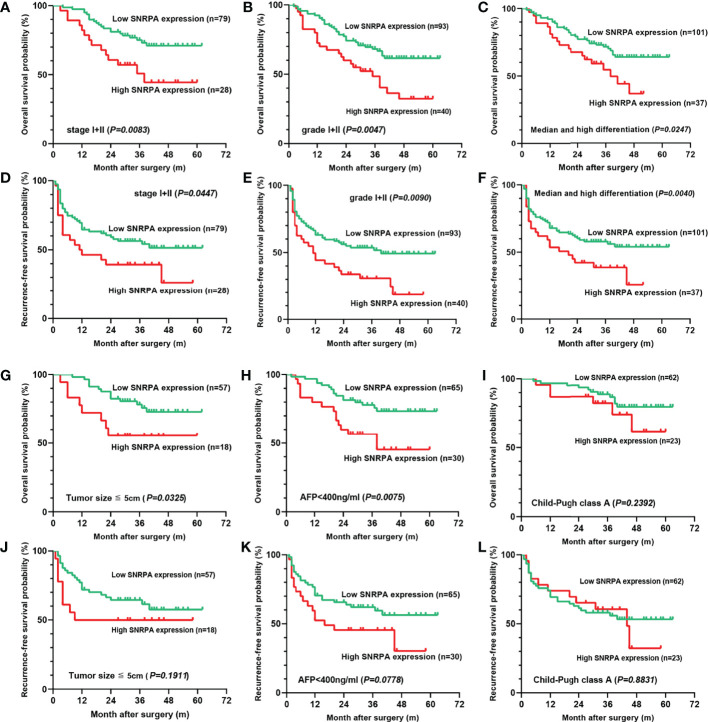
The prognostic value of the SNRPA protein levels in patients with early-stage subgroups. **(A–C)** In stage I+II **(A)**, grade I+II **(B)**, and median/high differentiation subgroups **(C)**, higher SNRPA protein expression significantly correlated with shorter OS. **(D–F)** In stage I+II **(D)**, grade I+II **(E)**, and median/high differentiation subgroups **(F)**, higher SNRPA protein expression significantly correlated with shorter RFS. **(G, H)** In tumor size ≤ 5cm **(G)**, AFP ≤400 ng/ml **(H)** subgroups, higher SNRPA protein expression correlated with poor OS. **(I)** There was no correlation between the SNRPA protein expression and OS in the Child-Pugh class A subgroup. **(J–L)** In tumor size ≤ 5cm **(J)**, AFP ≤400 ng/ml **(K)**, and Child-Pugh class A **(L)** subgroup, RFS did not significantly differ between the high and low SNRPA protein expression groups.

Further, we studied the prognostic significance of the SNRPA protein expression in early-stage (I+II), low tumor grade (grade I+II), low AFP level (≤400 ng/ml), tumor size ≤ 5cm, Child-Pugh class A, and median/high differentiation subgroups. The result indicated that in stage I+II, grade I+II, and median/high differentiation subgroups, both OS and RFS of patients with high SNRPA protein expression were significantly shorter than patients with low SNRPA protein expression (**Figures 3A-F**). In tumor size ≤ 5cm, AFP ≤400 ng/ml subgroups, high SNRPA protein expression correlated with poor OS (**Figures 3G, H**), but not correlated with RFS (**Figures 3J, K**). In addition, in the Child-Pugh class A subgroup, both OS and RFS did not significantly differ between the high and low SNRPA protein expression groups (**Figures 3I, L**).

### *SNRPA* Regulates the Proliferation and Migration of HCC Cells

**Figure 4:**
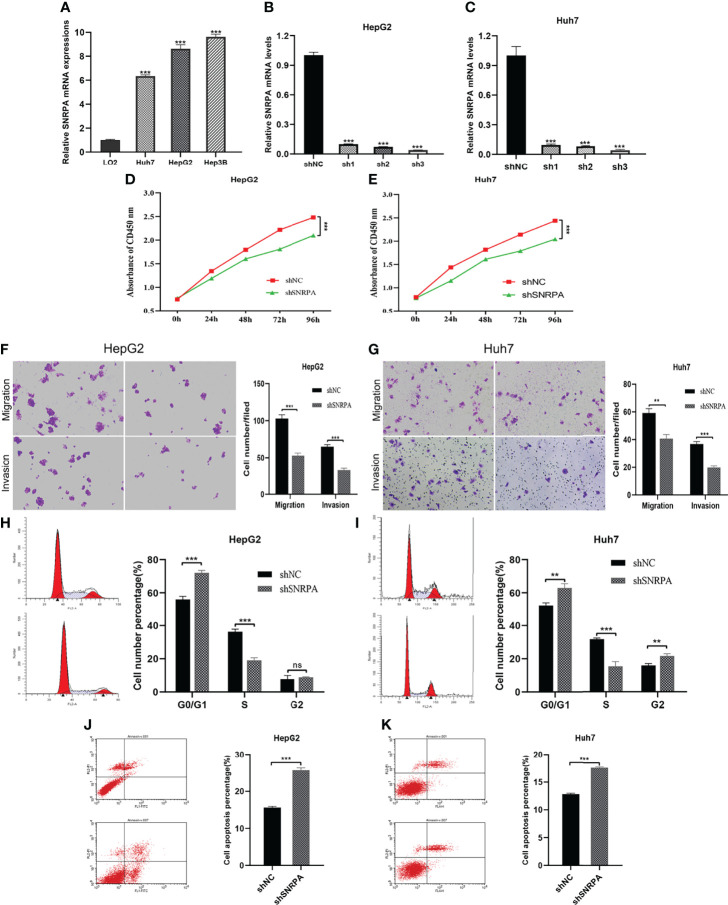
*SNRPA* regulates the proliferation and migration of HCC cells. **(A)** *SNRPA* mRNA expression was greater in HCC cells than in the normal liver cells. **(B, C)** The shSNRPA with lentiviral transfection were transfected into HepG2 **(B)** and Huh7 **(C)** cell lines. **(D, E)** Cell proliferation of HepG2 **(D)** and Huh7 **(E)** cells with shNC and shSNRPA were assessed by CCK-8 assays. **(F, G)** Representative images and quantified analysis of transwell assays in HepG2 **(F)** and Huh7 **(G)** cells with shNC and shSNRPA. **(H, I)** Cell cycle distribution of HepG2 **(H)** and Huh7 **(I)** cells with shNC and shSNRPA were assessed by flow cytometry. **(J, K)** Cell apoptosis rate of HepG2 **(J)** and Huh7 **(K)** cells with shNC and shSNRPA were determined by flow cytometry. **P < 0.01, ***P < 0.001. ns: no statistically significant.

We investigated the *SNRPA* expression in one normal liver cell line (LO2) and three HCC cells line (Huh7, HepG2, Hep3B) and found that *SNRPA* was significantly overexpressed in HCC cell lines (**Figure 4A**). Then, the targeted shRNAs (shSNRPA) with lentiviral transfection were transfected into HepG2 and Huh7 cell lines, respectively. Then, the targeted shRNAs (shSNRPA) with lentiviral transfection were transfected into HepG2 and Huh7 cell lines, respectively. The qRT-PCR validated that *SNRPA* mRNA expression was significantly inhibited in both cell lines. Due its maximum inhibitory effect in both cells, shSNRPA-3 was selected for subsequent experiments. (**Figures 4B, C**). The CCK-8 assays indicated that *SNRPA* knockdown inhibited the proliferation of both HepG2 and Huh7 cells compared to the shNC cells (**Figures 4D, E**). In addition, transwell assays demonstrated that shSNRPA significantly inhibited the cell migration and invasion in both HCC cells (**Figures 4F, G**). Furthermore, SNRPA knockdown markedly prolonged the G1 phase and shortened the S phase of both HepG2 and Huh7 cells (**Figures 4H, I**). Finally, flow cytometry detection illustrated that shSNRPA dramatically enhanced the apoptotic rate of both HCC cells (**Figures 4J, K**).

### Dysregulation of *SNRPA* Expression Correlated With DNA Methylation Status in Patients With HCC

**Figure 5:**
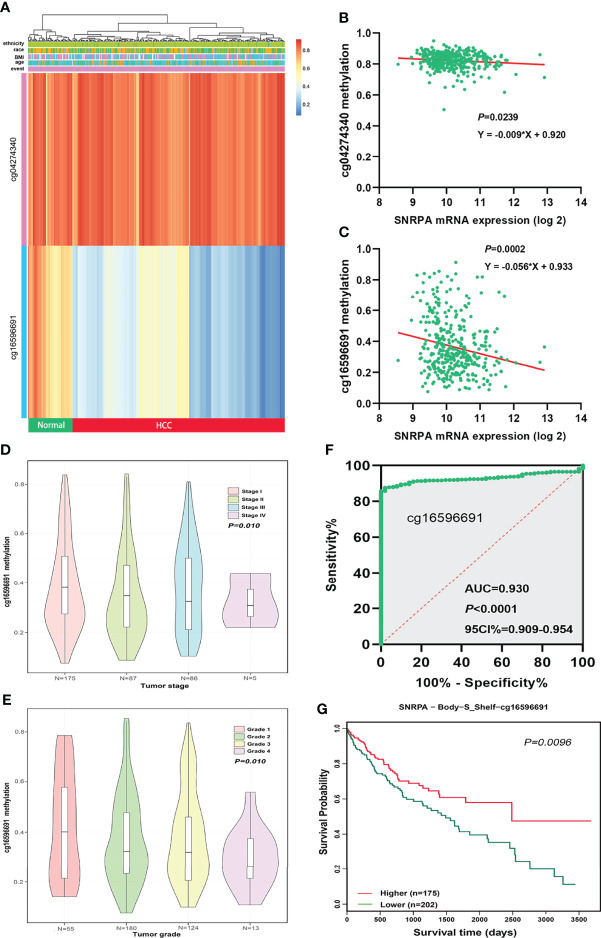
Aberrant DNA methylation level contributed to dysregulation of *SNRPA* expression in HCC patients. **(A)** The *SNRPA*-related methylated CpG sites in HCC was shown in the heat map. **(B, C)** The correlation between *SNRPA* mRNA expression and cg04274340 **(B)** and cg16596691 **(C)** methylation status. **(D, E)** The methylation status of cg16596691 gradually decreased as the tumor stage **(D)** and grade level increased **(E)**. **(F)** The prognostic significance of the methylation status of cg16596691 for HCC was assessed by the ROC curve. **(G)** Hypomethylation of cg16596691 correlated with poorer OS in HCC.

We investigated the correlations between *SNRPA* mRNA expression and DNA methylation status in the MethSurv database and identified two *SNRPA*-related methylation CpG sites in HCC: cg04274340 and cg16596691 (**Figure 5A**). We next analyzed Illumina Human Methylation 450 datasets in TCGA and found that *SNRPA* mRNA expression was significantly negatively related to the methylation status of the cg16596691 site (**Figures 5B, C**). In addition, the methylation status of cg16596691 gradually decreased as the tumor stage and grade level increased (**Figures 5D, E**). Moreover, ROC curve analysis revealed that the methylation status of cg16596691 exhibited excellent diagnostic significance for HCC in TCGA (AUC=0.930, P<0.001, 95% CI: 0.909-0.954, **Figure 5F**). The survival analysis also indicated that lower methylation of cg16596691 correlated with poorer overall survival probability in HCC (P=0.0096, **Figure 5G**). All results demonstrated that dysregulation of *SNRPA* expression and the poorer prognosis was associated with DNA methylation status in patients with HCC.

### Analysis of *SNRPA* Co-Expressed Genes in Patients With HCC

**Figure 6:**
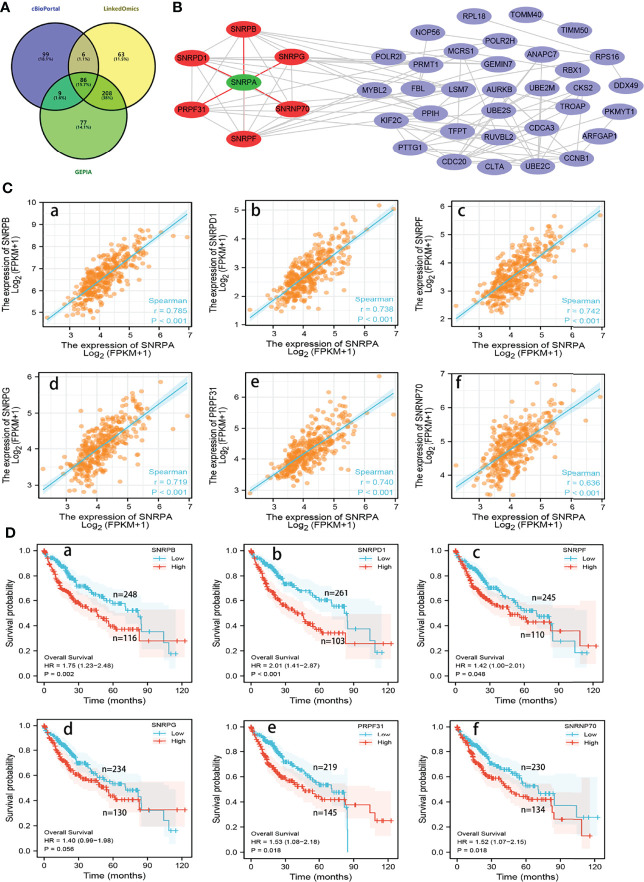
Analysis of co-expressed genes with *SNRPA* in HCC. **(A)** 86 overlapping co-expressed genes with *SNRPA* were identified from the three databases. **(B)** The PPI network showed that the SNRPD1, SNRPB, SNRPG, SNRPF, PRPF31, and SNRNP70 protein directly interact with SNRPA. **(C)** Correlation of *SNRPA* mRNA levels with *SNRPB* (a), *SNRPD1* (b), *SNRPF* (c), *SNRPG* (d), *PRPF31* (e), and *SNRNP70* (f) mRNA levels. **(D)** Associations between *SNRPB* (a), *SNRPD1* (b), *SNRPF* (c), *SNRPG* (d), *PRPF31* (e), and *SNRNP70* (f) mRNA levels and the OS of HCC patients.

We subsequently investigated the genes that correlated with *SNRPA* in their expression from the LinkedOmics, cBioPortal and GEPIA databases, respectively. The 86 overlapping genes from the three databases with Spearman’s values greater than 0.60 were selected as co-expressed genes with *SNRPA* (**Figure 6A**). Next, the Retrieval of Interacting Genes (STRING) database was utilized to analyze the significant interactions between SNRPA and its 86 co-expressed genes. We then established and visualized a protein-protein interaction (PPI) network with 40 nodes and 96 edges using Cytoscape software (**Figure 6B**). In the PPI network, six proteins (SNRPB, SNRPD1, SNRPG, SNRPF, PRPF31, SNRNP70) directly interacted with SNRPA. In TCGA database, Correlation analysis validated that these six genes significantly positively correlated with the expression of SNRPA (**Figure 6C**). Furthermore, the survival analysis in TCGA revealed that higher level of these six genes correlated with poorer overall survival probability in patients with HCC (**Figure 6D**).

### Overexpression of *SNRPA* Was Associated With the Spliceosome Signaling Pathway in HCC

**Figure 7:**
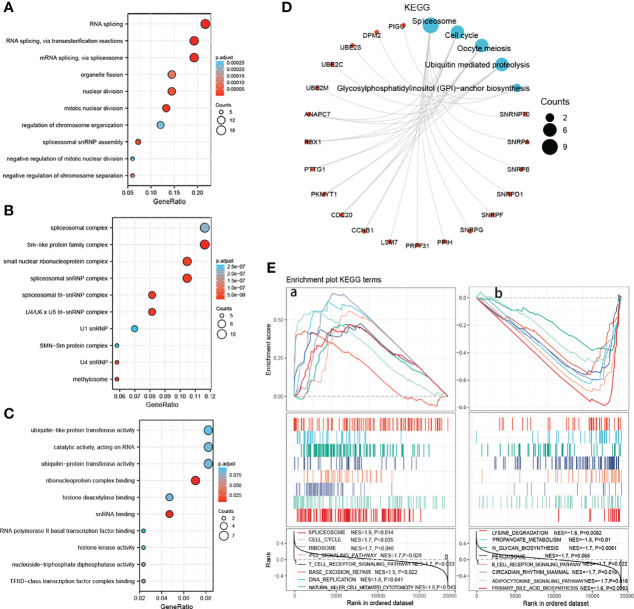
Functional enrichment analysis of *SNRPA* in HCC. **(A–C)** The bubble diagram for the biological process **(A)**, cellular component **(B)**, and molecular function **(C)** data in GO analysis. **(D)** The KEGG pathway enrichment analysis of co-expressed genes with *SNRPA*. **(E)** Identification of significant signaling pathways enriched in the high *SNRPA* mRNA expression patients **(A)** and low *SNRPA* mRNA expression patients **(B)** by GSEA.

For clarifying the mechanism and signaling pathway whereby *SNRPA* promotes the tumorigenesis and progression in HCC, we performed the GO and KEGG analyses on 86 co-expressed genes with *SNRPA* in the Database for Annotation, Visualization and Integrated Discovery (DAVID). In the GO Biological Process analysis, the co-expressed genes were mainly involved in splicing processes, such as RNA splicing, mRNA splicing *via* spliceosome, spliceosomal snRNP assembly (**Figure 7A**). In the GO cellular component, these genes were mainly related to spliceosomal complex, Sm-like protein family complex, spliceosomal snRNP complex, and spliceosomal tri-snRNP complex (**Figure 7B**). In the GO molecular function, these genes were mainly related to ubiquitin-like protein transferase activity, catalytic activity acting on RNA, ribonucleoprotein complex binding, and snRNA binding (**Figure 7C**). In the KEGG analysis, the Spliceosome, Cell cycle, Oocyte meiosis, Ubiquitin mediated proteolysis, Glycosylphosphatidylinositol (GPI)-anchor biosynthesis were greatly enriched (**Figure 7D**).

We next performed a GSEA using mRNA expression data in TCGA to further explore the potential pathways that *SNRPA* implicated in tumorigenesis and HCC progression. Results showed that high *SNRPA* level patients correlated with Spliceosome, Cell cycle, Ribosome, P53 signaling pathway, T cell receptor signaling pathway, Base excision repair, and DNA replication, etc (**Figures 7E, a**). Meanwhile, the low *SNRPA* level patients negatively correlated with Lysing degradation, Propanoate metabolism, N glycan biosynthesis, Peroxisome, B cell receptor signaling pathway, etc (**Figures 7E, b**). All results demonstrated that *SNRPA* mRNA was implicated in the spliceosome signaling pathway in HCC.

### Association Between *SNRPA* mRNA Expression and Tumor-Infiltrating Immune Cells

**Figure 8:**
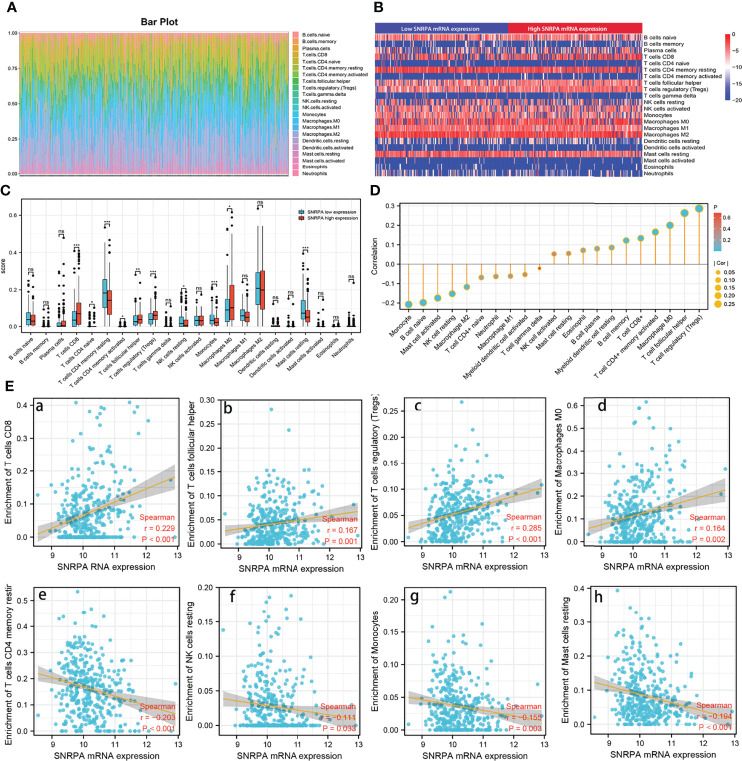
Association analysis of *SNRPA* mRNA expression and immune infiltration. **(A)** Estimation of fractions of immune cells of each tissue, where different colors represented different immune cells. **(B)** The heatmap showing the difference in immune cells infiltration between the high and low *SNRPA* expression group. **(C)** The comparison of estimated fractions of 22 immune cells between the high and low *SNRPA* expression group. **(D)** The correlation between *SNRPA* expression and 22 immune cells in HCC using ssGSEA with Spearman r analysis. **(E)** The correlation of *SNRPA* mRNA expression with immune infiltration level of **(a)** T cells CD8, **(b)** T cells follicular helper, **(c)** T cells regulatory, **(d)** Macrophages M0, **(e)** T cells CD4 memory restir, **(f)** NK cells resting, **(g)** Monocytes, **(h)** Mast cells resting. ns, no statistically significant.

Previous studies have shown that independent tumor-infiltrating lymphocytes play an essential role in the prognosis prediction in a variety of cancers (18–20). Therefore, we investigated the associations between *SNRPA* expression and 22 immune-cell subsets in HCC using the CIBERSORT algorithm and ssGSEA. We calculated the estimated fractions of 22 immune cells in each HCC tissue and visualized it in a box plot. As shown in **Figure 8A**, the different colors correspond to different types of immune cells (**Figure 8A**). Then, we investigated the infiltration difference of immune cells between the high and low *SNRPA* expression groups (**Figure 8B**). In addition, the CIBERSORT algorithm revealed that high *SNRPA* expression patients have a higher proportion of CD8+T cells, T cells follicular helper, T cells regulatory, Macrophages M0, whereas a lower proportion of T cells CD4+ memory resting, NK cells resting, Monocytes, and Mast cells resting (**Figure 8C**). We further investigated the correlation between SNRPA and 22immune cells in HCC using ssGSEA with Spearman r analysis (**Figure 8D**). Results uncovered that *SNRPA* mRNA expression positively correlated with infiltration levels of CD8+ T cells (r=0.229, P<0.001), T cells follicular helper (r=0.167, P=0.001), T cells regulatory (r=0.258, P<0.001) and Macrophages (r=0.164, P=0.002) (**Figures 8E a–d**). Whereas, *SNRPA* mRNA expression negatively correlated with infiltration levels of T cells CD4+ memory restir (r=-0.203, P<0.001), NK cells resting (r=-0.111, P=0.033), Monocytes (r=-0.155, P=0.003) and Mast cells resting (r=-0.194, P<0.001) (**Figures 8E e–h**). These results suggested that *SNRPA* potential regulates the extent of immune cell infiltration in HCC.

## Discussion

As one of the most common aggressive tumors, HCC is the fifth leading cause of cancer-related death worldwide and result in a significant economic and health burden (1, 21). Despite the great progress has been made in molecular targeted therapy and immunotherapy in the past several years, the overall prognosis of patients with HCC is still poor (17, 22–24). The low detection rate, rapid progression, and easy recurrence after curative hepatectomy were the primary causes of the poor prognosis in patients with HCC. Therefore, there is an urgent need for clarification of the molecular mechanism of the tumorigenesis and development of the HCC and identification of biomarkers with prognostic value and exploration of new targets to improve prognosis (25).

Spliceosome, was responsible for the excision of introns from pre-messenger RNA and the connection of exons in sequence (26–28). The splicing process of pre-messenger RNA was essential in gene expression in eukaryotic cells and the alterations of splicing patterns may lead directly to tumorigenesis (27, 29, 30). Numerous previous studies have shown that many spliceosomal relevant biomarkers can be used as the easy and non-invasive way to detect and estimate the prognosis of HCC. Splicing factor 3B subunit 1 (*SF3B1*) was reported overexpressed in HCC, and its silence inhibited cell viability, proliferation and migration of HCC cell lines (31). Small nuclear ribonucleoprotein polypeptide C (*SNRPC*), coding essential protein of spliceosome, was reported to promote the viability and epithelial-mesenchymal transition of HCC cells (32). In addition, Wang et al. reported that small nuclear ribonucleoprotein polypeptide D1 (*SNRPD1*) was regulated directly by miR-100 and promoted the progression of HCC through regulating the mTOR pathway and autophagy (33). Previous studies have confirmed that *SNRPA* was considered a tumor enhancer that can promote the tumorigenesis and progression of lung cancer, gastric cancer, cervical cancer and colorectal cancer (14, 15, 34–36). Herein, we first systematically investigated the prognostic value, clinical significance and gene function of *SNRPA* in HCC.

We investigated the *SNRPA* mRNA levels in HCC based on the TCGA and GEO database and found that *SNRPA* mRNA was significantly upregulated in HCC compared with normal tissues. In addition, *SNRPA* mRNA levels gradually increased with the tumor stage and tumor grade increased. ROC curve exhibited the excellent diagnostic significance of *SNRPA* mRNA level. Kaplan-Meier survival analyses revealed that patients with high *SNRPA* expression had shorter OS and RFS. Furthermore, in patients with early tumor stages (stage I+II) and grades (grade I+II), *SNRPA* mRNA levels still exhibited significant prognostic value. We next investigated the SNRPA protein levels in a cohort with 161 HCC patients and found that SNRPA protein also overexpressed in HCC samples compared with adjacent normal liver tissues. Moreover, higher SNRPA protein expression was statistically associated with poorer clinicopathological features and prognosis. Most importantly, multivariate cox analysis validated that SNRPA protein expression was an independent prognostic factor for HCC. These results illustrated that *SNRPA* was of good prognostic value in HCC. Therefore, postoperative SNRPA IHC detection may be helpful for the prediction of prognosis after HCC resection.

In our study, we also conducted cellular and molecular biology assays to further investigate the role of *SNRPA* in the progression of HCC and found that *SNRPA* mRNA was significantly upregulated in HCC cell lines compared with the normal liver cell line. When *SNRPA* was knockdown, the proliferative capacity of HepG2 and Huh7 cells were markedly inhibited. The transwell assay demonstrated that knockdown of the *SNRPA* also inhibited the migration and invasion of two HCC cells. In addition, knockdown of the *SNRPA* expression also inhibited cell cycle progression and promoted apoptosis. These results suggested that *SNRPA* plays prominent cancer-promoting roles and may be valuable drug targets.

DNA methylation, the most in-depth studied epigenetic alterations mechanism, has been confirmed to be causally related to various mechanisms of proliferation and metastasis of HCC (37). Growing evidence indicated that aberrant DNA methylation level extensively leads to the dysregulation of gene expression (38, 39). In our study, we found a clear correlation between the *SNRPA* overexpression and the hypomethylation of the CpG site cg16596691. Furthermore, the cg16596691 methylation level gradually decreased as the tumor stage and grade level increased, and hypomethylation of the CpG site cg16596691 was associated with poor OS in patients with HCC. Moreover, ROC curve analysis exhibited excellent diagnostic value of cg16596691 methylation status for HCC. These results demonstrated that hypomethylation of *SNRPA* contributed to *SNRPA* mRNA overexpression in HCC.

For clarifying the mechanism and signaling pathway whereby *SNRPA* promotes the tumorigenesis and progression in HCC, we performed the functional enrichment analysis. Results of GO Biological Process analysis revealed that *SNRPA* was mainly associated with the process of RNA splicing. In addition, KEGG and GSEA analysis revealed that *SNRPA* was involved in the spliceosome signaling pathway. Interestingly, numerous studies have shown that spliceosome signaling contributed enormously to the tumorigenesis and advancement of multiple tumor types (27, 40, 41). More importantly, in our previous study, the genes most relevant to survival were mainly enriched in the spliceosome pathway in the GEPIA database (40). Based on the above research results, it is reasonable to speculate that *SNRPA* functions as an oncogene that promotes HCC tumorigenesis and progression *via* the spliceosome signaling pathway, which needs further study.

Besides, we investigated the associations between *SNRPA* expression and 22 immune-cell subsets in HCC using the CIBERSORT algorithm and ssGSEA. The results of the CIBERSORT algorithm uncovered that high *SNRPA* expression correlated with a higher proportion of CD8+T cells, T cells follicular helper, T cells regulatory, Macrophages M0, and a lower proportion of T cells CD4 memory resting, NK cells resting, Monocytes, and Mast cells resting. Wolf et al. reported that high proportion of CD8+T cells of HCC promotes liver damage and HCC tumorigenesis and progression through interaction with NKT cells (42). In addition, the proliferation in T cells follicular helper was found in HCC compared with healthy control and promoted the progression (43). In our study, the high *SNRPA* expression level was associated with a lower proportion of NK cells resting and Monocytes. Previous studies have indicated that monocyte performs functions that contribute to antitumoral immunity, including phagocytosis, secretion of tumoricidal mediators (44, 45). Therefore, dysregulation of these immune cells in patients with higher *SNRPA* expression levels may be a reasonable explanation for the poor prognosis.

## Conclusion

SNRPA mRNA and protein levels were significantly greater in HCC tissues than in adjacent normal liver tissues and SNRPA could be an independent prognostic marker for predicting poor outcomes in HCC. The aberrant expression of SNRPA may be correlated with the dysregulation of DNA methylation. SNRPA plays prominent cancer-promoting roles through the spliceosome signaling pathway and may be valuable drug target. These findings can serve as a reference for subsequent experimental studies.

## Data Availability Statement

The datasets presented in this study can be found in online repositories. The names of the repository/repositories and accession number(s) can be found in the article/supplementary material.

## Ethics Statement

This study was performed according to the relevant medical ethics regulations and approved by the Human Research Ethics Committee of 900 Hospital of the Joint Logistics Team (Fuzhou, China). The patients/participants provided their written informed consent to participate in this study.

## Author Contributions

ZX and LL conceived and designed the research. YZ, XW and HW collected the data and provided study materials. YZ, XW and HW analysed and interpreted data. YZ, XW and HW and YJ operated supplementary experiment. YZ, XW and HW drafted the manuscript, then ZX and LL reviewed the manuscript. All authors contributed to the article and approved the submitted version.

## Funding

This work was supported by General Science and Technology Program of Jiangxi Provincial Health and Family Planning Commission (No. 20185004) for LL.

## Conflict of Interest

The authors declare that the research was conducted in the absence of any commercial or financial relationships that could be construed as a potential conflict of interest.

## Publisher’s Note

All claims expressed in this article are solely those of the authors and do not necessarily represent those of their affiliated organizations, or those of the publisher, the editors and the reviewers. Any product that may be evaluated in this article, or claim that may be made by its manufacturer, is not guaranteed or endorsed by the publisher.
